# Longitudinal Effects of Snow-season Activity Decline on Physical Function in Older Adults in Rural Snowy Regions

**DOI:** 10.1298/ptr.25-E10393

**Published:** 2026-04-28

**Authors:** Tomohito TADAISHI, Hideki SUZUKI

**Affiliations:** Graduate School of Rehabilitation Sciences, Health Sciences University of Hokkaido, Japan

**Keywords:** Light-intensity physical activity, Snowy cold region, Rural area, Tri-axial accelerometer, Snowy season

## Abstract

**Objectives:**

Physical activity levels vary seasonally, but the short-term impact of intensity-specific activity changes on older adults’ physical function remains unclear. This study aimed to examine seasonal changes in physical activity and their effects on physical function among older adults in a rural, snowy region.

**Methods:**

Thirty-nine older adults from Tobetsu-cho, Hokkaido, Japan, were included. Body composition and physical function were assessed during the non-snowfall season (June–September 2023) and 1 year later (June–September 2024). Accelerometers measured step counts and activity levels during both the non-snowfall and snowfall seasons (January–February 2024). Participants were grouped by the extent of change in activity levels (greater-change vs. smaller-change). Two-way analysis of variance (ANOVA) assessed differences in 1-year changes in physical function.

**Results:**

Repeated-measures 2-way ANOVA showed a significant main effect of season on body fat percentage, appendicular skeletal muscle mass index, grip strength, and walking speed in the light-intensity physical activity (LPA) category. A significant interaction was observed for the Five Times Sit-to-Stand Test (FTSST). Bonferroni’s test revealed a significant worsening of FTSST scores (*p* <0.01) after 1 year, but only in the group with a greater LPA decline.

**Conclusions:**

A greater decrease in LPA during the snowfall was associated with short-term declines in lower-limb function, as measured by FTSST. Given its link to fall risk and loss of independence, maintaining LPA during snowy seasons may be essential for maintaining lower-limb function in older adults.

## Introduction

The global population aged 65 years or older was approximately 600 million in 2019 and is projected to triple to 1.6 billion by 2050, accounting for 20% of the world’s total population^1)^. In addition, the number of people aged 80 years or older increased from 126 million to 447 million, highlighting growing interest in healthy aging^1)^. The risk of developing sarcopenia and frailty increases with age^2,3)^, and if unaddressed, these conditions also raise the likelihood of requiring long-term care^4,5)^.

Physical activity levels are associated with both frailty and sarcopenia^6,7)^, and the World Health Organization recommends increasing physical activity even among the elderly^8)^. Physical activity is influenced by both individual and external environmental factors. Climate, in particular, affects physical activity levels^9)^, with studies reporting decreases in step count and overall physical activity during snowy seasons in rural, cold regions^10)^.

Not only step count and moderate-to-vigorous physical activity (MVPA) but also light-intensity physical activity (LPA) are significantly reduced during the snowy season^10)^. These seasonal declines in activity are believed to impair physical function, even in the short term.

A previous study examining seasonal changes in step count and physical function over 1 year reported that maintaining step count during the snowy season positively affected short-term changes in walking speed^11)^. However, prior studies have methodological limitations. Using December—which already falls within the snowy season—as a baseline and February—the peak of midwinter—as the comparison may not accurately capture seasonal variations. Moreover, these studies focused solely on step count, without accounting for seasonal changes in overall physical activity levels.

Given that step counts are highly correlated with MVPA but weakly correlated with LPA, they may underestimate physical activity related to household chores and other low-intensity tasks^12)^. Therefore, it is essential to examine the effects of sedentary behavior (SB), LPA, and MVPA on physical function over 1 year, accounting for seasonal changes in these parameters. This analysis should use data from clearly defined seasonal periods—namely, the non-snowfall season (June–September) and the snowfall season (January–February)—to more accurately capture seasonal variations. This approach is crucial for assessing the longitudinal impact of seasonal fluctuations in SB, LPA, and MVPA on physical function in older adults.

Among older adults, LPA—primarily reflecting non-exercise physical activity and weakly associated with both step count and MVPA—decreases substantially during the snowfall season^10)^. Clarifying whether this seasonal decline in LPA affects physical function, even over a short period, may provide insights for developing effective care prevention strategies in cold and snowy regions.

This study focused on participants who completed a 1-year follow-up and examined the potential causal relationship between seasonal changes in physical activity during the snowfall season (exposure) and subsequent changes in body composition and physical function (outcomes).

## Methods

### Participants

This study was conducted among older adults residing in Tobetsu Town, Ishikari District, Hokkaido, Japan—a rural region characterized by a snowy and cold climate^13)^. The Tobetsu Elderly Health Support Project, conducted from June to September 2023, initially enrolled 74 participants. Of these, 55 individuals aged 65 years or older were included in the preliminary analysis after excluding those with incomplete or inadequate data.

The final analysis focused on a subset of participants who demonstrated continued engagement throughout the study—specifically, the 39 individuals who also participated in the winter survey (January–February 2024) and the follow-up survey (June–September 2024). Participant flow is visually represented in Figure 1.

**Fig. 1.**
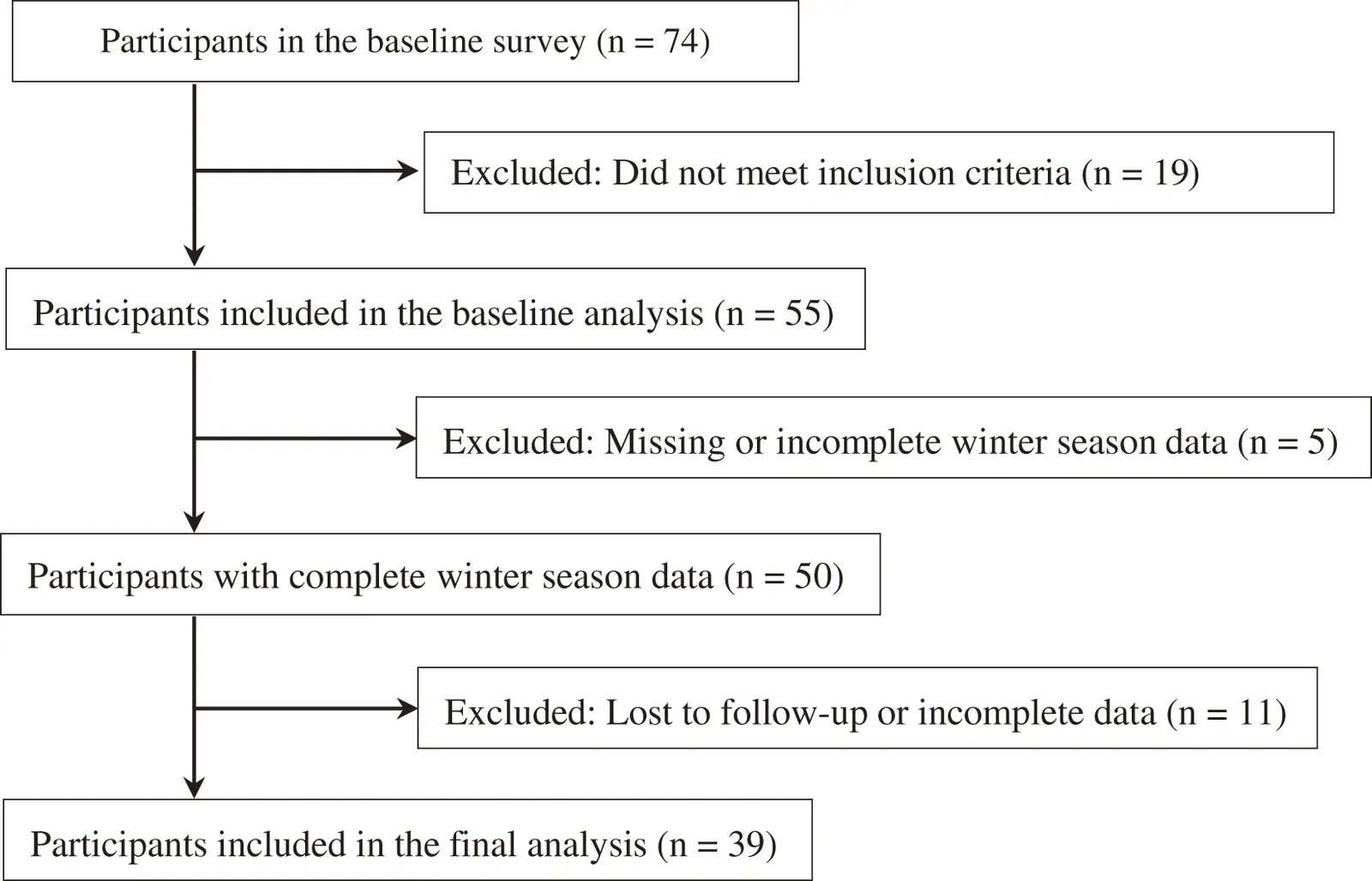
Flowchart of participant selection.

### Data collection and climate conditions during the snowfall season

Data collection during the snowfall season was carried out in January and February 2024. During this period, local meteorological records showed mean temperatures ranging from −4.2°C to −4.0°C and maximum snow depths of 127–138 cm. These records highlight the severe winter environment in which the participants’ physical activity was monitored, contrasting with the non-snowfall baseline^13)^.

### Measurement items

During the non-snowfall season (June–September 2023), a questionnaire collected demographic and health information, including age, sex, presence of cohabiting family members, and medical history. Body composition was assessed via body mass index (BMI), body fat percentage, and appendicular skeletal muscle mass index (ASMI) using an Inbody S10 (InBody Japan, Tokyo, Japan). Physical function was evaluated using grip strength and the Short Physical Performance Battery (SPPB)^14,15)^. Comfortable walking speed was calculated from the SPPB 4-m walk test, defined as walking path (4 m) divided by the walking time (seconds). Additionally, the time required to complete the Five Times Sit-to-Stand Test (FTSST) was recorded in seconds.

Home-based physical activity was assessed using a tri-axial accelerometer (Active Style Pro HJA-750C; Omron, Kyoto, Japan). Participants wore the device during waking hours for 7 consecutive days, excluding bathing or water immersion over a monitoring period to ensure compliance. It was positioned on the right/left anterior side of the waist, and participants recorded daily wear and removal times using a standardized form. To prevent behavioral modification, the device displayed only the time of day. Its validity has been previously established^16)^, and physical activity intensity was recorded in 60-s epochs.

Physical activity during the snowfall season (January–February 2024) was measured using a tri-axial accelerometer in older adults who had participated at baseline. For both the seasons, data were considered valid if the device was worn for ≥10 h/day on at least 4 days^17)^. Periods of zero activity counts exceeding 90 min were classified as non-wear time^18)^. Mean values across all valid measurement days were used for each participant.

Wearing time and step count were recorded during both the non-snowfall and snowfall seasons. Time spent in SB (≤1.5 metabolic equivalents [METs]), LPA (1.6–2.9 METs), and MVPA (≥3.0 METs) was calculated. A “break” was defined as an LPA lasting more than 1 min following a >1 min of SB^19)^.

A follow-up assessment was conducted 1 year after baseline (June–September 2024). Demographic and medical information were collected using the same procedures. Physical measurements included height, weight, BMI, body fat percentage, ASMI, grip strength, and SPPB^14,15)^. Comfortable walking speed (m/s) was again calculated from the SPPB 4-m walk test, and FTSST time was also recorded in seconds.

### Group classification

Changes in SB, LPA, and MVPA between the baseline non-snowfall and snowfall seasons were calculated. Based on the median values of these changes, participants were classified into 2 groups: greater-change group and smaller-change group. The median values used for classification were: +39.9 min for SB, –58.5 min for LPA, and –5.5 min for MVPA.

### Statistical analysis

An a priori power analysis using G*Power software (version 3.1.9.2, University of Düsseldorf, Germany) indicated that a minimum sample size of 34 participants was required to achieve a statistical power (1–β) of 0.80 for a repeated-measures 2-way ANOVA, assuming a medium effect size (f = 0.25) and an alpha level of 0.05. As 39 participants were included in the final analysis, the study was designed to have adequate power to detect interaction effects.

Normality of each dependent variable was assessed using the Shapiro–Wilk test. Changes in body composition and SPPB scores from baseline to 1 year were analyzed using either the paired t-test or the Wilcoxon signed-rank test, depending on whether the data were normally distributed.

Body composition indicators (BMI, body fat percentage, and ASMI) and physical function measures (grip strength, comfortable walking speed, and FTSST) were treated as dependent variables. Seasonal changes (time: baseline and 1 year) and group differences based on changes in physical activity intensity (SB, LPA, and MVPA) were analyzed using repeated-measures 2-way analysis of variance (ANOVA). Variables that violated the normality assumption were log-transformed prior to ANOVA.

When a significant interaction effect was detected, simple main effects were examined using Bonferroni-adjusted multiple comparisons. Baseline demographic differences between groups were assessed using an independent t-test for age and a chi-squared (χ^2^) test for sex ratios.

All analyses were performed using SPSS Statistics 26 (IBM, Tokyo, Japan), and statistical significance was set at p <0.05.

### Research ethics

This study adhered to the principles of the Declaration of Helsinki and utilized data only from participants who provided informed consent following written and verbal explanations. The study was approved by the Research Ethics Committee of the Health Sciences University of Hokkaido (23R179233).

## Results

The mean age of participants was 80.2 years, and 23 (59%) were female (Table 1). Approximately 95% lived with family members, and 44% had a history of hypertension. Details on cohabitation status and pre-existing medical conditions are presented in Table 1.

**Table 1.** Participant characteristics and physical function at baseline and 1-year follow-up

Variable	Baseline (n = 39)	1 Year follow-up (n = 39)	p Value
Age (years)	80.2 (6.4), 81.0 [77.0–84.0]	—	
Sex (male/female), n	16/23	—	
Living with others, n	37 (94.9%)	—	
Osteoarticular disease, n	7 (17.9%)	7 (17.9%)	
Neurological disease, n	0 (0.0%)	0 (0.0%)	
Heart disease, n	3 (7.7%)	3 (7.7%)	
Respiratory disease, n	1 (2.6%)	1 (2.6%)	
Hypertension, n	17 (43.6%)	17 (43.6%)	
Diabetes, n	8 (20.5%)	8 (20.5%)	
Height (cm)	156.1 (9.7), 153.0 [150.0–165.0]	—	
Weight (kg)	56.2 (9.5), 57.0 [48.0–63.0]	56.1 (9.8), 58.0 [47.0–63.0]	0.678^†^
BMI (kg/m^2^)	23.1 (3.8), 22.5 [20.4–25.1]	23.2 (3.8), 22.5 [20.4–25.2]	0.639^‡^
Body fat percentage (%)	28.2 (8.3), 27.6 [21.8–33.4]	29.4 (8.3), 29.1 [23.8–34.8]	0.002^†^
ASMI (kg/m^2^)	6.49 (0.89), 6.60 [5.8–7.17]	6.34 (0.88), 6.40 [5.8–7.0]	0.003^†^
SPPB total score	12.0 [11.0–12.0]	12.0 [11.0–12.0]	0.024^‡^
SPPB balance score	4.0 [4.0–4.0]	4.0 [3.0–4.0]	0.306^‡^
SPPB walking speed score	4.0 [4.0–4.0]	4.0 [4.0–4.0]	0.125^‡^
SPPB chair stand score	4.0 [4.0–4.0]	4.0 [4.0–4.0]	0.063^‡^

Mean (standard deviation), median [interquartile range], n (%).

^†^Paired t-test.

^‡^Wilcoxon signed-rank test.

BMI, body mass index; ASMI, appendicular skeletal muscle mass index; SPPB, short physical performance battery

No significant changes were observed in body weight or BMI, with mean values remaining nearly identical from baseline to the 1-year follow-up. In contrast, the average body fat percentage increased by 1.2%, and ASMI decreased by 0.15 kg/m^2^ (Table 1). Regarding physical performance, although the median total SPPB score and interquartile range remained unchanged at 12 [11–12], the Wilcoxon signed-rank test indicated a significant difference (p <0.05). This result reflects an imbalance in individual-level changes, with 13 participants showing a decrease in their SPPB scores, whereas 4 participants showed an increase.

Seasonal changes in step counts and physical activity showed an average decrease of 1135 steps per day, with a median decrease of 837 steps (Table 2). SB increased by approximately 40 min/day. LPA decreased by an average of 70 min and a median of 60 min/day. MVPA decreased by an average of 10 min and a median of 5 min/day (Table 2).

**Table 2.** Seasonal differences in step count and physical activity variables

Variable	Non-snowfall season	Snowfall season	Change
Wear time (min/day)	859.5 (102.7), 849.0 [778.9–934.6]	822.5 (81.7), 810.4 [776.2–886.0]	−36.9 (67.2), −18.9 [−64.7 to 12.8]
Step count (steps/day)	3710.0 (2626.0), 2710.0 [1934.4–5167.6]	2574.6 (1761.5), 2209.1 [1241.0–3393.3]	−1135.4 (1803.2), −836.8 [−1347.5 to −7.1]
SB (min/day)	486.6 (113.2), 491.0 [401.0–558.7]	533.1 (90.6), 526.4 [482.9–570.4]	46.6 (90.9), 39.9 [−23.1 to 114.0]
LPA (min/day)	341.6 (83.6), 352.1 [271.6–413.0]	268.8 (70.0), 276.1 [226.9–322.3]	−72.8 (63.5), −58.5 [−125.3 to −19.6]
MVPA (min/day)	31.4 (26.8), 24.0 [11.7–37.0]	20.6 (16.8), 14.0 [9.6–24.9]	−10.8 (20.8), −5.5 [−20.1 to 0.3]
Number of breaks (times/day)	62.5 (13.8), 64.1 [49.8–71.7]	57.9 (14.8), 59.6 [47.9–66.1]	−4.6 (11.5), −3.0 [−7.8 to 2.1]

Mean (standard deviation), median [interquartile range].

SB, sedentary behavior; LPA, light-intensity physical activity; MVPA, moderate-to-vigorous physical activity

Participants were grouped into greater- vs. smaller-change categories based on median changes in number of steps, SB, LPA, and MVPA. Changes from baseline to 1 year were analyzed using repeated-measures 2-way ANOVA.

For the SB-based classification, a significant group effect was observed for comfortable walking speed, along with significant seasonal effects for body fat percentage, ASMI, grip strength, and walking speed (Table 3).

**Table 3.** Associations between seasonal changes in physical activity intensity and physical function (n = 39)

Variable	Group	Baseline	1 Year	Group effect, F value(p)	Time effect, F value(p)	Interaction, F value(p)
(a) Sedentary behavior-based grouping						
Age (years)	Greater-change (n = 19)	79.1 (6.0)	—	—	—	—
	Smaller-change (n = 20)	81.3 (6.5)	—			
Body fat (%)	Greater-change	28.2 (8.4)	29.8 (7.9)	0.036 (p = 0.851)	11.283 (p = 0.002)	0.996 (p = 0.325)
	Smaller-change	28.1 (8.3)	29.0 (8.8)			
ASMI (kg/m^2^)	Greater-change	6.5 (0.9)	6.30 (0.9)	0.023 (p = 0.879)	9.893 (p = 0.003)	0.274 (p = 0.604)
	Smaller-change	6.5 (0.9)	6.4 (0.9)			
Grip strength (kgf)	Greater-change	24.8 (7.9)	25.7 (6.2)	0.135 (p = 0.715)	5.181 (p = 0.029)	0.425 (p = 0.518)
	Smaller-change	26.1 (8.3)	26.6 (7.9)			
Comfortable walking speed (m/s)	Greater-change	1.05 (0.19)	0.98 (0.21)	4.540 (p = 0.040)	11.965 (p = 0.001)	0.013 (p = 0.911)
	Smaller-change	1.21 (0.24)	1.10 (0.23)			
FTSST (s)	Greater-change	9.9 (3.5)	10.4 (3.1)	2.762 (p = 0.105)	1.670 (p = 0.204)	0.256 (p = 0.616)
	Smaller-change	8.6 (2.6)	8.8 (2.5)			
(b) Light-intensity physical activity-based grouping						
Age (years)	Greater-change (n = 19)	79.4 (6.7)	—	—	—	—
	Smaller-change (n = 20)	80.9 (5.9)	—			
Body fat (%)	Greater-change	29.1 (7.9)	30.6 (7.6)	0.658 (p = 0.422)	11.219 (p = 0.002)	0.829 (p = 0.369)
	Smaller-change	27.3 (8.6)	28.2 (8.8)			
ASMI (kg/m^2^)	Greater-change	6.4 (0.8)	6.2 (0.8)	0.510 (p = 0.480)	9.885 (p = 0.003)	0.028 (p = 0.869)
	Smaller-change	6.6 (1.0)	6.4 (0.9)			
Grip strength (kgf)	Greater-change	26.6 (8.3)	27.4 (6.8)	1.190 (p = 0.709)	5.110 (p = 0.030)	0.141 (p = 0.709)
	Smaller-change	24.4 (7.9)	24.8 (8.0)			
Comfortable walking speed (m/s)	Greater-change	1.15 (0.25)	1.05 (0.26)	0.070 (p = 0.793)	12.095 (p = 0.001)	0.145 (p = 0.706)
	Smaller-change	1.11 (0.21)	1.03 (0.19)			
FTSST (s)	Greater-change	8.7 (3.7)	9.9 (3.8)	0.553 (p = 0.462)	2.164 (p = 0.150)	7.620 (p = 0.009)
	Smaller-change	9.8 (2.4)	9.3 (1.8)			
(c) Moderate-to-vigorous physical activity-based grouping						
Age (years)	Greater-change (n = 19)	79.7 (7.9)	—	—	—	—
	Smaller-change (n = 20)	80.6 (4.4)	—			
Body fat (%)	Greater-change	27.8 (7.7)	29.4 (6.9)	0.017 (p = 0.895)	11.427 (p = 0.002)	0.138 (p = 0.248)
	Smaller-change	28.5 (9.0)	29.3 (9.5)			
ASMI (kg/m^2^)	Greater-change	6.4 (0.8)	6.3 (0.9)	0.353 (p = 0.556)	9.890 (p = 0.003)	0.013 (p = 0.911)
	Smaller-change	6.6 (0.9)	6.4 (0.9)			
Grip strength (kgf)	Greater-change	24.6 (8.0)	25.0 (6.4)	0.748 (p = 0.393)	5.023 (p = 0.031)	0.186 (p = 0.669)
	Smaller-change	26.3 (8.2)	27.3 (7.6)			
Comfortable walking speed (m/s)	Greater-change	1.11 (0.25)	1.02 (0.28)	0.781 (p = 0.382)	11.976 (p = 0.001)	0.001 (p = 0.978)
	Smaller-change	1.16 (0.20)	1.05 (0.17)			
FTSST (s)	Greater-change	9.7 (3.7)	10.5 (3.7)	1.517 (p = 0.226)	1.708 (p = 0.119)	0.656 (p = 0.423)
	Smaller-change	8.8 (2.4)	8.8 (1.7)			

Mean (standard deviation), 2-way repeated measures ANOVA was performed to evaluate the main effects of group and time, as well as interaction effects.

Grip strength, gait speed, and FTSST were log-transformed prior to analysis.

ASMI, appendicular skeletal muscle mass index; FTSST, Five Times Sit-to-Stand Test; ANOVA, analysis of variance

Using the LPA-based classification, similar seasonal effects were observed. In addition, a significant season × group interaction was identified for the FTSST (Table 3).

Likewise, the MVPA-based classification showed significant seasonal effects for the same physical measures (Table 3).

Given the significant interaction between FTSST and season in the LPA group, Bonferroni's multiple comparison test was conducted. Results indicated that FTSST was significantly higher 1 year after baseline only in the greater-change group (*p* <0.01).

There were no significant differences in age or sex ratio between groups.

## Discussion

This study examined the relationship between seasonal changes in step counts and objectively measured physical activity, and subsequent changes in skeletal muscle mass and physical function over 1 year among older adults in a rural area. During the snowfall season, the median number of steps, LPA time, and MVPA decreased to approximately 82%, 79%, and 58%, respectively, while SB time increased to approximately 107% compared to the non-snowfall season.

However, the increase in SB time did not fully correspond to the decrease in overall activity. This discrepancy may be due to a roughly 4% reduction in device wear time during the snowfall season. It is likely that part of the decrease in LPA and MVPA was replaced by non-wear time.

Regarding the activity data, the observed increase in SB was proportionally smaller than the decrease in LPA and MVPA. This pattern may be explained by seasonal changes in daily rhythms. A previous study reported that older adults wake up significantly later in winter than in summer, while bedtime remains largely unchanged^20)^. In our study region, sunrise occurs approximately 3 h later at the winter solstice (7:00 a.m.) than at the summer solstice (4:00 a.m.)^21)^. Because sleep duration was not directly measured in this study, delayed wake-up times may have shortened daily device wear time during the snowfall season. Consequently, truncation of the measurable active period may account for the smaller-than-expected increase in SB relative to the reductions in other activity intensities.

Maintaining 7000–8000 steps per day and at least 15–20 min of MVPA daily represents a critical threshold for preserving skeletal muscle mass in older adults^6)^. In this study, many participants did not meet these thresholds even during the non-snowfall season, and this shortfall increased during the snowfall season. The mean participant age was 80.2 years—approximately 8 years older than in the previous study^6)^—and their average ASMI was about 0.28 kg/m^2^ lower. These characteristics suggest that many participants were at high risk for muscle mass decline, which may have contributed to lower step counts and MVPA.

Regardless of seasonal variation in physical activity, ASMI decreased by approximately 0.15 kg/m^2^ (−2.3%) 1 year after the snowfall season, exceeding the −1% annual decline reported in previous studies^22)^. In this study, the average daily step count during the non-snowfall season was approximately 3700 steps (median: 2700 steps), which decreased by about 1135 steps (median: 837 steps) during the snowfall season. These results indicate that overall activity levels, as reflected by annual step counts, remained below the threshold required to maintain skeletal muscle mass^6)^, and that ASMI declined irrespective of the degree of seasonal variation.

MVPA time was not normally distributed. The median MVPA duration during the non-snowfall season was 24 min, exceeding the recommended threshold, whereas it declined to 14 min during the snowfall season, falling below the standard^6)^. Consistent with the low daily step counts, this reduction in MVPA may have contributed to the observed decline in ASMI, regardless of the magnitude of seasonal change.

An increase in body fat percentage was also observed after 1 year, which may be associated with reduced energy expenditure due to decreased physical activity during the snowfall season. Furthermore, age-related declines in basal metabolic rate and skeletal muscle mass may also contribute to fat accumulation. The coexistence of decreased skeletal muscle mass and increased fat mass is referred to as sarcopenic obesity, a condition linked to atherosclerosis and increased cardiovascular risk^23)^. Among older adults with low step counts and limited physical activity, it is important to monitor both skeletal muscle loss and potential fat gain.

Regarding physical function, changes in grip strength, comfortable walking speed, and FTSST were assessed over 1 year. There was a significant main effect of time on grip strength and comfortable walking speed, with changes observed 1 year after the snowfall season, regardless of the extent of seasonal variation in physical activity. Although grip strength slightly increased in both groups, the change was less than 1 kg. Previous studies have shown that age-related declines in muscle strength are more pronounced in the lower than in the upper limbs^24)^, suggesting grip strength may have been preserved over the relatively short duration of this study.

A significant main effect of time was also observed for comfortable walking speed, with both groups exhibiting a decline after 1 year, regardless of the magnitude of seasonal variation in physical activity. A previous study in older adults reported that maintaining step counts during the snowfall season positively affected walking speed 1 year later^11)^; however, the present findings suggest that the overall level of physical activity in this population may have been insufficient to prevent a decline in walking performance.

That study measured maximum walking speed, reporting values between 1.71 and 1.75 m/s^11)^. In contrast, the present study found walking speeds ranging from 0.98 to 1.21 m/s, likely due to different measurement methods: the previous study measured maximum walking speed, while the present study assessed walking speed using the walking component of the SPPB^14)^, which evaluates the time required to walk at a comfortable pace.

Moreover, the previous study categorized participants into 2 groups based on changes in step count between early winter and midwinter: those who maintained or increased their step count and those whose step count decreased. In contrast, the present study evaluated participants according to the magnitude of change in physical activity, as most participants experienced reductions in activity levels during the snowfall season. This methodological difference in assessing activity changes may have influenced the results.

Furthermore, in the previous study, step counts were 5466 at baseline and 5735 during the snowfall season, exceeding 5000 steps in both periods. In contrast, the median step counts in the present study were 2710 at baseline and 2209 during the snowfall season, both well below 3000 steps. These findings suggest that participants in the present study were older and engaged in lower levels of physical activity than those in the previous study, which may have contributed to the observed decline in walking speed, irrespective of the magnitude of seasonal variation in physical activity.

An interaction effect was observed in FTSST after 1 year when classified by the amount of change in LPA, with a significant increase in FTSST time in the group with a greater seasonal change in LPA. Older adults living in rural areas of snowy and cold regions showed the greatest seasonal reduction in LPA, with a decrease of approximately 1 h/day during the snowfall season. This suggests that a greater decline in LPA may negatively impact FTSST after 1 year. Although MVPA is recommended even for older adults^8)^, attention should also be directed toward the decline in LPA during the snowfall season.

Recent studies indicate that LPA alone can reduce the risk of functional decline and is beneficial for depressive symptoms and life expectancy^25–27)^. However, evidence linking total LPA to deterioration in physical function remains limited^28,29)^. Existing studies have primarily involved individuals with knee osteoarthritis or chronic pain, with inconsistent findings^28,29)^.

In this context, Chen et al. conducted a 6-year follow-up study on older adults living in a suburban area of Fukuoka City, Japan^27)^. During the follow-up, 16.2% of participants developed functional disability. The study found a linear association between the total amount of MVPA and functional disability risk. Moreover, even short bouts of LPA were beneficial^27)^. Functional disability was defined based on a new certification for long-term care insurance. Although this definition does not differentiate between disability due to inactivity and that due to disease, the study remains the only one to demonstrate a preventive effect of LPA on the development of functional disability^27)^.

However, the study was conducted in a region without snowfall, where winter temperatures did not drop below freezing^27)^. Physical activity at baseline was measured over a week between May and August, and seasonal changes in physical activity were not assessed.

In this study, we found that a decrease in LPA during the snowfall season—an aspect not emphasized in previous research—had an impact on physical function after 1 year. It is well established that the number of older adults capable of engaging in MVPA decreases with age^30)^, and our findings indicate that MVPA comprised only about 3% of total activity time during the non-snowfall season and approximately 2% during the snowfall season. This suggests that preventing seasonal decline in LPA during snowy periods may help mitigate short-term declines in physical function.

In particular, FTSST, a predictor of future falls and functional decline^31,32)^, was affected. These findings highlight the need for strategies to prevent reductions in LPA during snowy seasons to support care prevention efforts in cold and snowy regions.

This study has several limitations. First, although the sample size was determined a priori based on a power analysis, the number of participants was relatively limited, which may have reduced the ability to detect small effect sizes. Second, because accelerometer monitoring was conducted during winter, the measurement period included days with snowfall; however, incomplete 7-day data for some participants prevented a uniform characterization of weather conditions for the days included in the final analysis.

## Conclusions

Older adults who experienced a greater decrease in LPA during the snowfall season showed a significant increase in FTSST scores after 1 year, indicating a decline in lower-limb function. In contrast, those with a smaller decrease in LPA did not exhibit such changes. These findings suggest that seasonal variations in physical activity, particularly reductions in LPA during the snowfall season, may negatively impact physical function in older adults living in snowy and cold regions. Interventions aimed at minimizing the decline in LPA during winter may be important for maintaining functional ability and preventing disability in this population.

## References

[1] He W, Goodkind D, et al.: *An aging world: 2015*. International population reports, P95/16–1. Washington (DC): U.S. Census Bureau; 2016.

[2] Kojima G, Iliffe S, et al.: Prevalence of frailty in Japan: a systematic review and meta-analysis. J Epidemiol. 2017; 27: 347–353. 10.1016/j.je.2016.09.008 PMC5549151 28142044

[3] Makizako H, Nakai Y, et al.: Prevalence of sarcopenia defined using the Asia working group for sarcopenia criteria in Japanese community-dwelling older adults: a systematic review and meta-analysis. Phys Ther Res. 2019; 22: 53–57. 10.1298/ptr.R0005 PMC6992522 32015941

[4] Yeung SSY, Reijnierse EM, et al.: Sarcopenia and its association with falls and fractures in older adults: a systematic review and meta-analysis. J Cachexia Sarcopenia Muscle. 2019; 10: 485–500. 10.1002/jcsm.12411 PMC6596401 30993881

[5] Osuka Y, Kim H, et al.: Sarcoscore: A novel approach for assessing sarcopenia and functional disability in older adults. J Clin Med. 2020; 9: 692. 10.3390/jcm9030692 PMC7141295 32143446

[6] Park H, Park S, et al.: Yearlong physical activity and sarcopenia in older adults: the Nakanojo Study. Eur J Appl Physiol. 2010; 109: 953–961. 10.1007/s00421-010-1424-8 20336310

[7] Steffl M, Bohannon RW, et al.: Relationship between sarcopenia and physical activity in older people: a systematic review and meta-analysis. Clin Interv Aging. 2017; 12: 835–845. 10.2147/CIA.S132940 PMC5441519 28553092

[8] Bull FC, Al-Ansari SS, et al.: World Health Organization 2020 guidelines on physical activity and sedentary behaviour. Br J Sports Med. 2020; 54: 1451–1462. 10.1136/bjsports-2020-102955 PMC7719906 33239350

[9] Amagasa S, Inoue S, et al.: Changes in rural older adults’ sedentary and physically-active behaviors between a non-snowfall and a snowfall season: compositional analysis from the NEIGE study. BMC Public Health. 2020; 20: 1248. 10.1186/s12889-020-09343-8 PMC7433168 32807133

[10] Tadaishi T, Hasegawa J, et al.: Seasonal and sex differences in instrumental activities of daily living and objective physical activity among older adults residing in rural areas with snow and cold regions. J Rural Med. 2025; 20: 1–12. 10.2185/jrm.2024-025 PMC11704604 39781303

[11] Mizumoto A, Ihira H, et al.: Physical activity during winter in old-old women associated with physical performance after one year: a prospective study. Curr Gerontol Geriatr Res. 2015; 2015: 25345. 10.1155/2015/253451 PMC4491546 26185495

[12] Inaba R, Yamakawa S, et al.: Measurement of various intensities of physical activities and categorization of “Locomotive” and “Household” activities provide a subject-specific detailed assessment. Sci Rep. 2021; 11: 22104. 10.1038/s41598-021-99392-9 PMC8585886 34764435

[13] Hokkaido Tobetsu: Past weather observation data. https://www-town-tobetsu-hokkaido-jp.translate.goog/soshiki/kensetsu/45044.html?_x_tr_sl=ja&_x_tr_tl=en&_x_tr_hl=ja. (Accessed January 11, 2026).

[14] Guralnik JM, Simonsick EM, et al.: A short physical performance battery assessing lower extremity function: association with self-reported disability and prediction of mortality and nursing home admission. J Gerontol. 1994; 49: M85–M94. 10.1093/geronj/49.2.m85 8126356

[15] Mijnarends DM, Meijers JM, et al.: Validity and reliability of tools to measure muscle mass, strength, and physical performance in community-dwelling older people: a systematic review. J Am Med Dir Assoc. 2013; 14: 170–178. 10.1016/j.jamda.2012.10.009 23276432

[16] Ohkawara K, Oshima Y, et al.: Real-time estimation of daily physical activity intensity by a triaxial accelerometer and a gravity-removal classification algorithm. Br J Nutr. 2011; 105: 1681–1691. 10.1017/S0007114510005441 21262061

[17] Mâsse LC, Fuemmeler BF, et al.: Accelerometer data reduction: a comparison of four reduction algorithms on select outcome variables. Med Sci Sports Exerc. 2005; 37: S544–S554. 10.1249/01.mss.0000185674.09066.8a 16294117

[18] Heesch KC, Hill RL, et al.: Validity of objective methods for measuring sedentary behaviour in older adults: a systematic review. Int J Behav Nutr Phys Act. 2018; 15: 119. 10.1186/s12966-018-0749-2 PMC6260565 30477509

[19] Owen N, Healy GN, et al.: Too much sitting: the population health science of sedentary behavior. Exerc Sport Sci Rev. 2010; 38: 105–113. 10.1097/JES.0b013e3181e373a2 PMC3404815 20577058

[20] Hashizaki M, Nakajima H, et al.: A longitudinal large-scale objective sleep data analysis revealed a seasonal sleep variation in the Japanese population. Chronobiol Int. 2018; 35: 933–945. 10.1080/07420528.2018.1443118 29589960

[21] Ephemeris Computation Office, National Astronomical Observatory of Japan: Local Calendar. https://eco.mtk.nao.ac.jp/koyomi/dni/index.html.en (Accessed May 18, 2025)

[22] Mitchell WK, Williams J, et al.: Sarcopenia, dynapenia, and the impact of advancing age on human skeletal muscle size and strength; a quantitative review. Front Physiol. 2012; 3: 260. 10.3389/fphys.2012.00260 PMC3429036 22934016

[23] Kohara K: Sarcopenic obesity in aging population: current status and future directions for research. Endocrine. 2014; 45: 15–25. 10.1007/s12020-013-9992-0 23821364

[24] Lynch NA, Metter EJ, et al.: Muscle quality. I. Age-associated differences between arm and leg muscle groups. J Appl Physiol(1985). 1999; 86: 188–194. 10.1152/jappl.1999.86.1.188 9887130

[25] Jakicic JM, Kraus WE, et al.: Association between bout duration of physical activity and health: systematic review. Med Sci Sports Exerc. 2019; 51: 1213–1219. 10.1249/MSS.0000000000001933 PMC6527142 31095078

[26] Yasunaga A, Shibata A, et al.: Cross-sectional associations of sedentary behaviour and physical activity on depression in Japanese older adults: an isotemporal substitution approach. BMJ Open. 2018; 8: e022282. 10.1136/bmjopen-2018-022282 PMC6169747 30257848

[27] Chen T, Honda T, et al.: Dose-response association between accelerometer-assessed physical activity and incidence of functional disability in older Japanese adults: a 6-year prospective study. J Gerontol A Biol Sci Med Sci. 2020; 75: 1763–1770. 10.1093/gerona/glaa046 PMC7494030 32134454

[28] Dunlop DD, Song J, et al.: Relation of physical activity time to incident disability in community dwelling adults with or at risk of knee arthritis: prospective cohort study. BMJ. 2014; 348: g2472. 10.1136/bmj.g2472 PMC4004786 24782514

[29] Makino K, Lee S, et al.: Daily physical activity and functional disability incidence in community-dwelling older adults with chronic pain: a prospective cohort study. Pain Med. 2019; 20: 1702–1710. 10.1093/pm/pny263 30561732

[30] Jansen FM, Prins RG, et al.: Physical activity in non-frail and frail older adults. PLoS One. 2015; 10: e0123168. 10.1371/journal.pone.0123168 PMC4409218 25910249

[31] Zhang F, Ferrucci L, et al.: Performance on five times sit-to-stand task as a predictor of subsequent falls and disability in older persons. J Aging Health. 2013; 25: 478–492. 10.1177/0898264313475813 23407343

[32] Makizako H, Shimada H, et al.: Predictive cutoff values of the Five-Times Sit-to-Stand Test and the Timed “Up & Go” Test for disability incidence in older people dwelling in the community. Phys Ther. 2017; 97: 417–424. 10.2522/ptj.20150665 28371914

